# Extremely Low-Frequency Electromagnetic Fields Affect the miRNA-Mediated Regulation of Signaling Pathways in the GC-2 Cell Line

**DOI:** 10.1371/journal.pone.0139949

**Published:** 2015-10-06

**Authors:** Yong Liu, Wen-bin Liu, Kai-jun Liu, Lin Ao, Jia Cao, Julia Li Zhong, Jin-yi Liu

**Affiliations:** 1 Institute of Toxicology, College of Preventive Medicine, Third Military Medical University, Chongqing, China; 2 College of Bioengineering, Chongqing University, Chongqing, China; National Research Council, ITALY

## Abstract

Extremely low-frequency electromagnetic fields (ELF-EMFs) can affect male reproductive function, but the underlying mechanism of this effect remains unknown. miRNA-mediated regulation has been implicated as an important epigenetic mechanism for regulatory pathways. Herein, we profiled miRNA expression in response to ELF-EMFs *in vitro*. Mouse spermatocyte-derived GC–2 cells were intermittently exposed to a 50 Hz ELF-EMF for 72 h (5 min on/10 min off) at magnetic field intensities of 1 mT, 2 mT and 3 mT. Cell viability was assessed using the CCK–8 assay. Apoptosis and the cell cycle were analyzed with flow cytometry. miRNA expression was profiled using Affymetrix Mouse Genechip miRNA 3.0 arrays. Our data showed that the growth, apoptosis or cell cycle arrest of GC–2 cells exposed to the 50 Hz ELF-EMF did not significantly change. However, we identified a total of 55 miRNAs whose expression significantly changed compared with the sham group, including 19 differentially expressed miRNAs (7 miRNAs were upregulated, and 12 were downregulated) in the 1 mT exposure group and 36 (9 miRNAs were upregulated, and 27 were downregulated) in the 3 mT exposure group. The changes in the expression of 15 selected miRNAs measured by real-time PCR were consistent with the microarray results. A network analysis was used to predict core miRNAs and target genes, including miR-30e-5p, miR-210-5p, miR-196b-5p, miR-504-3p, miR-669c-5p and miR-455-3p. We found that these miRNAs were differentially expressed in response to different magnetic field intensities of ELF-EMFs. GO term and KEGG pathway annotation based on the miRNA expression profiling results showed that miRNAs may regulate circadian rhythms, cytokine-cytokine receptor interactions and the p53 signaling pathway. These results suggested that miRNAs could serve as potential biomarkers, and the miRNA-mediated regulation of signaling pathways might play significant roles in the biological effects of ELF-EMFs.

## Introduction

Humans are surrounded by power supply lines as well as many household and commercial devices, and exposure to extremely low-frequency electromagnetic fields (ELF-EMFs) is increasing. The potential of ELF-EMFs to exert harmful biological effects on human health is of increasing concern [1–3]. The male reproductive system is more sensitive to ELF-EMFs. Many studies have reported that ELF-EMFs can decrease semen quality in animals and humans by impairing the motility of and inducing morphometric abnormalities in spermatozoa and by increasing oxidative stress [4–6]. However, other studies have found that occupational exposure or short-term exposure to a 50 Hz ELF-EMF does not adversely affect spermatogenesis [7]. Prenatal exposure to ELF-EMFs did not increase miscarriages or induce gross external, skeletal or visceral malformations when fields up to 20 mT were tested [8]. Because of such contradictory results, the potential hazards of ELF-EMFs exposure remain unclear, and convincing evidence is lacking. Therefore, elucidating the potential effects of ELF-EMFs on the male reproductive system is crucial.

MicroRNAs, a group of endogenous small non-coding RNAs (19~25 nucleotides), are expressed in almost all biota, including animals, viruses and plants [9, 10]. Their primary biological function is the regulation of gene expression at the post-transcriptional level, mainly via binding to the 3’-untranslated region of target genes [11, 12]. The binding of miRNAs to their target mRNAs may inhibit the translation or enhance the degradation of the target mRNAs [13]. miRNA-mediated regulation has been implicated as an important epigenetic mechanism for regulatory pathways that are linked to various human cancers and male reproductive disorders [14–16]. Increasing evidence indicates that miRNAs are required for spermatogenesis and male fertility, and cumulative research has shown that miRNA-mediated regulation contributes to the etiology of testicular germ cell tumors [17].

Aberrant miRNA expression has been related to the development of human germ cell tumors, but little is known about effect of ELF-EMFs on miRNA expression. ELF-EMFs may epigenetically modify cells, which may account for the adverse effects of ELF-EMFs on the male reproductive system. Considering the biological role of miRNAs, we investigated the expression of miRNAs in the mouse spermatocyte-derived GC–2 cell line and examined whether some miRNAs could act as biomarkers of exposure to ELF-EMFs.

## Materials and Methods

### Cell culture

Mouse spermatocyte-derived GC–2 spd cells (GC–2 cells) were obtained from the American Tissue Culture Collection (ATCC, Rockville, MD, USA) and cultured in high-glucose DMEM (Hyclone, Logan, UT, USA) containing 10% fetal bovine serum at 37°C in a 5% CO_2_ humidified atmosphere.

### Exposure procedure

The exposure system was designed and provided by the Foundation for Information Technologies in Society (IT’IS foundation, Zurich, Switzerland) as described previously [18, 19]. Briefly, this system was designed for the testing of electromagnetic field exposures. The exposure system consists of a power frequency generator, an arbitrary function generator, a narrow band amplifier as well as two rectangular waveguides. The setup generated a vertical EMF, which composed of two four-coil systems (two coils with 56 windings, two coils with 50 windings), and was placed inside a metal chamber. The system was composed of two identical exposure chambers. One of the chambers was a sham-exposed and the other chamber was an exposed. Sham and exposure cell dishes were simultaneously placed into an incubator in which the environmental conditions were constant (37°C, 5% CO2). The exposure set-up was controlled and monitored by a computer through specific sensors, which can automatically control the exposure parameters including exposure intensity and exposure time. The temperature difference between sham and ELF-EMF exposure never exceeded 0.3°C. After overnight starvation, GC–2 cells were exposed to a 50 Hz ELF-EMF at magnetic intensities of 1 mT, 2 mT and 3 mT for 72 h (5 min on / 10 min off).

### Evaluation of cell viability

A cell counting kit–8 (CCK–8, Dojindo, Japan) was used to evaluate cell viability. Briefly, 3000 cells per well were seeded into 96-well plates in 100 μl of cell culture medium, and a 50 Hz ELF-EMF was then applied at different magnetic intensities for 72 h. The medium was subsequently mixed with 10 μl of the CCK–8 reaction solution and incubated for 1 h at 37°C. The optical density was measured at 450 nm using a microplate reader (SpectraMax M2; Molecular Devices, Sunnyvale, CA, USA). Five parallel wells were set up in each experiment. Each measurement was repeated three times.

### Detection of apoptosis and the cell cycle via flow cytometry

After ELF-EMF exposure, cell apoptosis was analyzed via flow cytometry using Annexin V-FITC and propidium iodide (PI) double-staining. To assess the cell cycle, GC–2 cells were harvested and fixed in 75% ice-cold ethanol overnight at 4°C. The fixed cells were washed twice with ice-cold PBS and stained with 50 mg/ml PI that contained 50 mg/ml RNase A (DNase free) for 30 min at 37°C. The cells were then analyzed in a flow cytometer. The experiments were conducted three times.

### Microarray analysis

The potential mechanism of miRNA expression was investigated and analyzed using Affymetrix Mouse Genechip miRNA 3.0 Arrays. The microarray experiments and data analysis were carried out at the Gminix company (Shanghai, China) following previously published methods [20]. Markedly changed miRNAs were designated as miRNAs that showed the highest fold changes, as verified by real-time PCR. Microarray data have been deposited in NCBI Gene Expression Omnibus with series accession number GSE71147.

### RNA extraction and real-time PCR

Following exposure to a 50 Hz ELF-EMF, the total RNA was extracted from the GC–2 cells using a TRIzol Reagent Kit (Invitrogen, USA). cDNAs were obtained with a reverse transcription PCR (RT-PCR) kit (Promega, USA). miRNA expression was examined using the Bio-Rad IQ5 Detection System and SYBR Green PCR Master mix (Promega, USA). Each real-time PCR amplification reaction (20 μl total volume) contained 2 μl of cDNA, 10 μl of 2× SYBR^®^ Green Real-time PCR Master Mix, 0.8 μl of each of the forward and reverse primer and 6.4 μl of ultrapure water. The samples were denatured by heating at 95°C for 20 sec, followed by 40 cycles of amplification (95°C for 10 sec, 60°C for 20 sec and 70°C for 6 sec). The expression levels of miRNAs were normalized to the expression level of U6 in each sample using the cycle threshold (Ct) method and the 2^-∆Ct^ formula. Each measurement was repeated three times and normalized against the sham group.

### Statistical analysis

All data were expressed as the means ± SD from at least three independent experiments that were performed in duplicate. The differences between the sham and ELF-EMF groups were analyzed using two-way ANOVA and Student’s t-test. Significant differences were established at P < 0.05.

## Results

### Effects of 50 Hz ELF-EMF exposure on the growth of GC–2 cells

**Fig 1:**
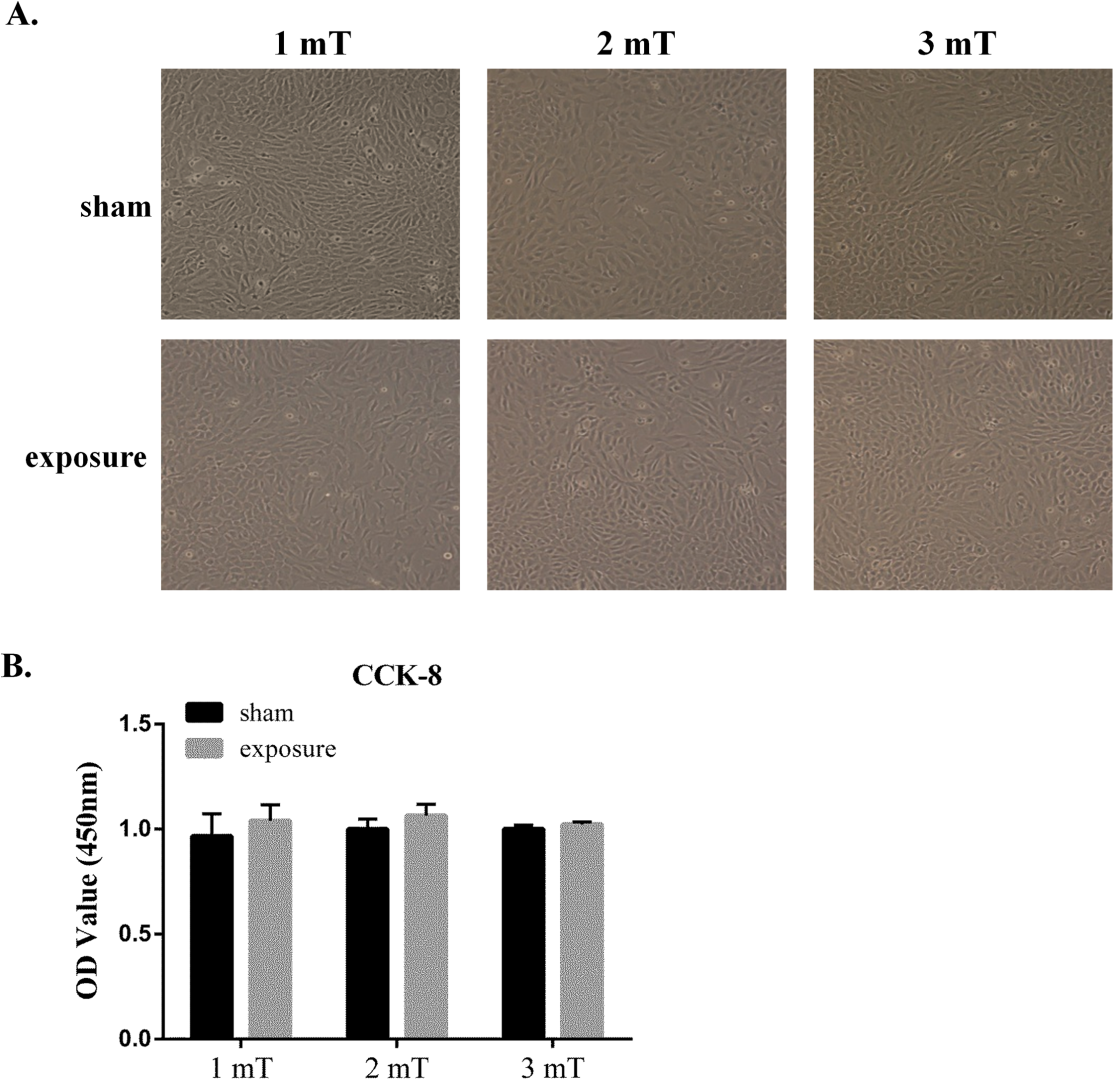
50 Hz ELF-EMF did not affect the growth of GC–2 cells. A. Representative images of the morphology of GC–2 cells exposed to a 50 Hz ELF-EMF at different magnetic intensities of 1 mT, 2 mT and 3 mT for 72 h. B. The 50 Hz ELF-EMF did not affect the proliferation of GC–2 cells. Cell viability was examined with the CCK–8 assay. These data are expressed as the mean ± SD of three independent experiments.

The cell viability was detected with the CCK–8 kit following 50 Hz ELF-EMF exposure at different magnetic intensities for 72 h to explore the effects of the ELF-EMF on the growth of GC–2 cells. Our results showed that the 50 Hz ELF-EMF did not markedly affect the morphology (Fig 1A) or viability (CCK–8) (Fig 1B) of GC–2 cells.

### Effects of 50 Hz ELF-EMF exposure on the apoptosis and cell cycle of GC–2 cells

**Fig 2:**
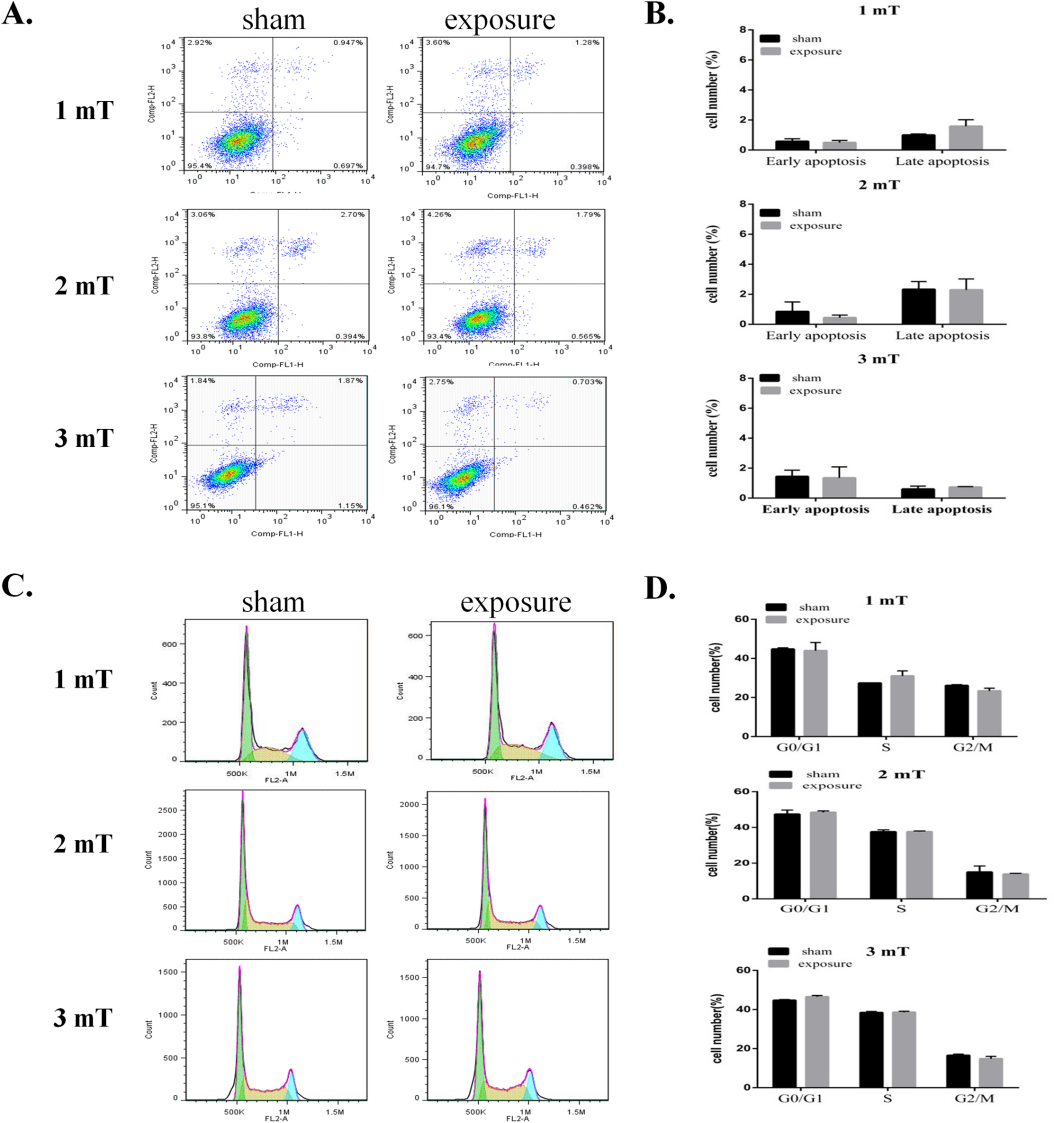
50 Hz ELF-EMF did not affect the apoptosis or cell cycle of GC–2 cells. A. Flow cytometry assays with Annexin V-FITC and PI double-staining of GC–2 cells. Representative dots plots for GC–2 cells at different magnetic intensities. B. Quantitative analysis of the apoptosis of GC–2 cells. C. GC–2 cells were stained with PI and analyzed with a flow cytometer. Representative results of the cell cycle analysis of GC–2 cells at different magnetic intensities. D. The percentages of cell cycle are represented with bar graphs. The data are expressed as the mean ± SD from three independent experiments.

To further confirm the effects of 50 Hz ELF-EMF exposure on GC–2 cell proliferation, the apoptosis and cell cycle of GC–2 cells after ELF-EMF exposure were analyzed with a flow cytometer. We found that 50 Hz ELF-EMF did not induce apoptosis in GC–2 cells compared with the sham group (Fig 2A and 2B). Additionally, the percentages of G1-, S-, and G2-phase cells did not significantly differ between the sham and exposure groups after treatment with 50 Hz ELF-EMF for 72 h (Fig 2C and 2D). These findings confirmed that 50 Hz ELF-EMF did not induce apoptosis or cell cycle arrest in GC–2 cells.

### Changes in miRNA expression induced by 50 Hz ELF-EMF exposure

**Fig 3:**
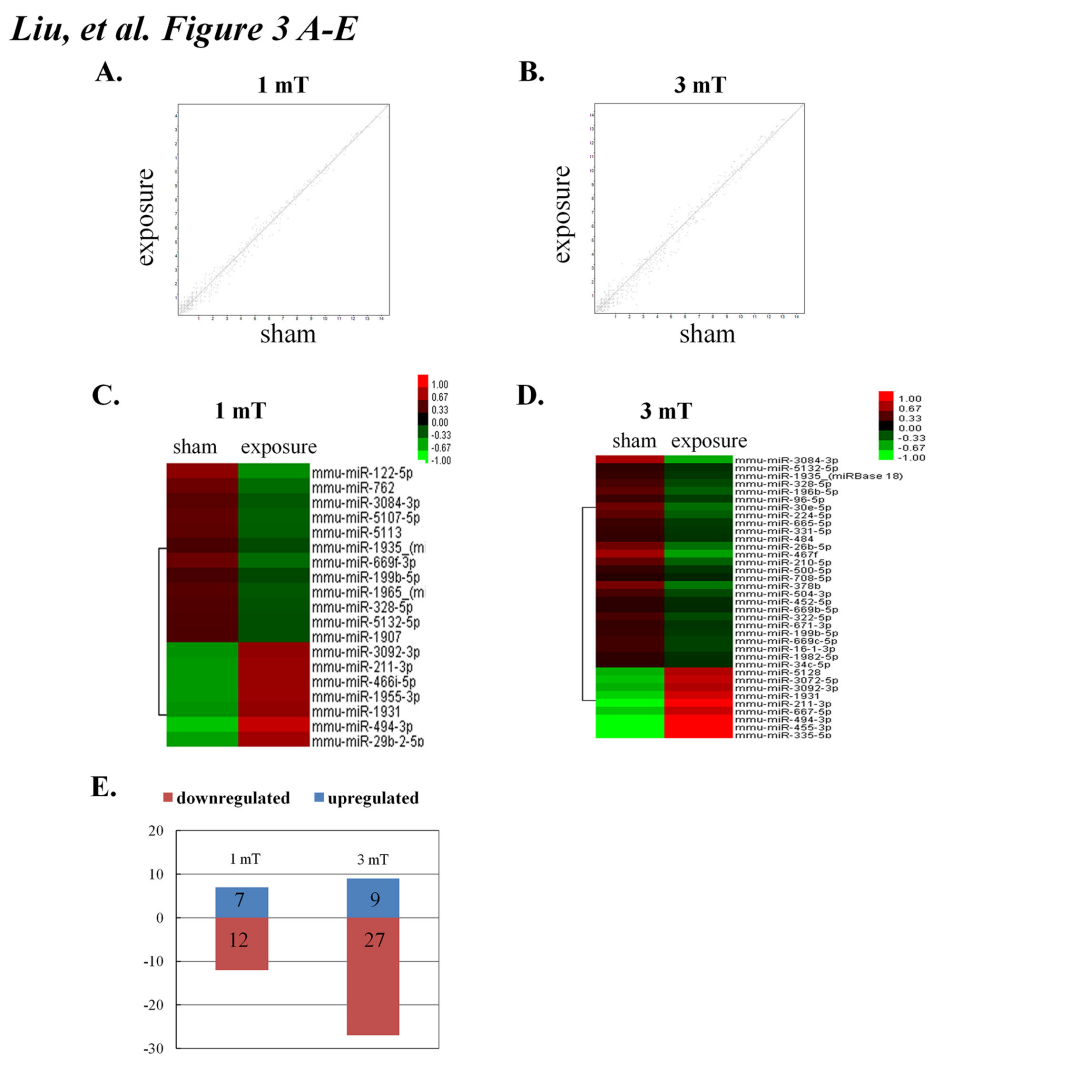
50 Hz ELF-EMF induced changes in miRNA expression. A. Scatter-plots for assessing the variations in miRNA expression in the GC–2 cell line between the sham and exposure groups at a magnetic field intensity of 1 mT. B. Scatter-plots for assessing the variations in miRNA expression in the GC–2 cell line between the sham and exposure groups at a magnetic field intensity of 3 mT. C. Hierarchical clustering of differentially expressed miRNAs in the GC–2 cell line between sham and exposure pass volcano plots at a magnetic field intensity of 1 mT. D. Hierarchical clustering of differentially expressed miRNAs in the GC–2 cell line between sham and exposure pass volcano plots at a magnetic field intensity of 3 mT. The color bar displaying the fluorescence intensity corresponds to the miRNA expression levels. Highly expressed miRNAs are shown in red, while those expressed at low levels are shown in green. E. The total number of miRNAs that were up- and downregulated after EMF exposure.

To identify miRNAs that were differentially expressed between the sham and ELF-EMF exposure groups, we performed an Affymetrix microarray analysis to establish the miRNA expression profiles. The total RNA was extracted from the sham and exposure groups. The data from each sample were globally normalized. Scatter-plots were employed to assess changes in the miRNA expression of GC–2 cells between the sham and exposure groups at 1 mT and 3 mT, as shown in Fig 3A and 3B, respectively. We identified 55 miRNAs whose expression markedly changed in response to ELF-EMF exposure (Fold change > 1.5), as shown in Fig 3C and 3D. Of the 19 differentially expressed miRNAs in the 1 mT exposure group, 7 miRNAs were upregulated, while 12 were downregulated. Of the 36 differentially expressed miRNAs in the 3 mT-treated groups, 9 miRNAs were upregulated, and 27 were downregulated (Fig 3E). We used real-time PCR to test and verify several differentially expressed miRNAs that were either downregulated or upregulated in GC–2 cells at a field intensity of 1 mT or 3 mT according to the Affymetrix arrays. The clustering of the differentially expressed miRNAs across the ELF-EMF exposure and sham groups is shown in Tables 1 and 2. The miRNAs that were most highly up- and downregulated at a magnetic field intensity of 1 mT were miR-494-3p (+2.3) and miR-122-5p (-2.6), while the most highly up- and downregulated miRNAs at a magnetic field intensity of 3 mT were miR-494-3p (+3.3) and miR-3084-3p (-3.7) (Tables 1 and 2). Interestingly, miR-494-3p was the most highly upregulated miRNA at a magnetic field intensity of 1 mT or 3 mT.

**Table 1:** miRNA expression changes in the GC–2 cell line after 72 h of exposure to 50 Hz power frequency electromagnetic radiation at an electric field intensity of 1.0 mT.

microRNA	Fold change	Chromosomal location
mmu-miR-122-5p	0.37	chr18
mmu-miR–762	0.45	chr7
mmu-miR-669f-3p	0.45	chr2
mmu-miR–5107	0.48	chr18
mmu-miR–5113	0.48	chr15
mmu-miR-3084-3p	0.49	chr19
mmu-miR–1965	0.50	chr7
mmu-miR-328-5p	0.52	chr8
mmu-miR–5132	0.52	chrX
mmu-miR–1907	0.53	chr15
mmu-miR–1935	0.53	chr11
mmu-miR-199b-5p	0.54	chr2
mmu-miR-3092-3p	1.80	chr3
mmu-miR–1931	1.82	chr10
mmu-miR-211-3p	1.88	chr7
mmu-miR-466i-5p	1.88	chr13
mmu-miR-1955-3p	1.89	chr2
mmu-miR-29b-2-5p	1.97	chr1
mmu-miR-494-3p	2.38	chr12

**Table 2:** miRNA expression changes in the GC–2 cell line after 72 h of exposure to 50 Hz power frequency electromagnetic radiation at an electric field intensity of 3.0 mT.

microRNA	Fold change	Chromosomal location
mmu-miR-3084-3p	0.27	chr19
mmu-miR-467f	0.28	chr11
mmu-miR-378b	0.35	chr11
mmu-miR-26b-5p	0.37	chr1
mmu-miR-30e-5p	0.37	chr4
mmu-miR-210-5p	0.40	chr7
mmu-miR-224-5p	0.41	chrX
mmu-miR-196b-5p	0.41	chr6
mmu-miR-322-5p	0.46	chrX
mmu-miR-504-3p	0.46	chrX
mmu-miR-328-5p	0.46	chr8
mmu-miR-16-1-3p	0.48	chr14
mmu-miR-669c-5p	0.48	chr2
mmu-miR-665-5p	0.49	chr10
mmu-miR-96-5p	0.49	chr6
mmu-miR-199b-5p	0.50	chr13
mmu-miR–1935	0.50	chr11
mmu-miR-331-5p	0.50	chr10
mmu-miR-500-5p	0.51	chrX
mmu-miR-671-3p	0.51	chr5
mmu-miR–484	0.52	chr16
mmu-miR–5132	0.52	chrX
mmu-miR-1982-5p	0.53	chr10
mmu-miR-34c-5p	0.53	chr9
mmu-miR-452-5p	0.53	chrX
mmu-miR-669b-5p	0.54	chr2
mmu-miR-708-5p	0.55	chr7
mmu-miR-3092-3p	1.80	chr3
mmu-miR–5128	1.81	chr2
mmu-miR-3072-5p	1.98	chr12
mmu-miR-667-5p	2.07	chr12
mmu-miR–1931	2.17	chr10
mmu-miR-211-3p	2.72	chr7
mmu-miR-455-3p	2.81	chr4
mmu-miR-335-5p	3.05	chr6
mmu-miR-494-3p	3.30	chr12

### qPCR confirmation of differentially expressed miRNAs following 50 Hz ELF-EMF exposure

**Fig 4:**
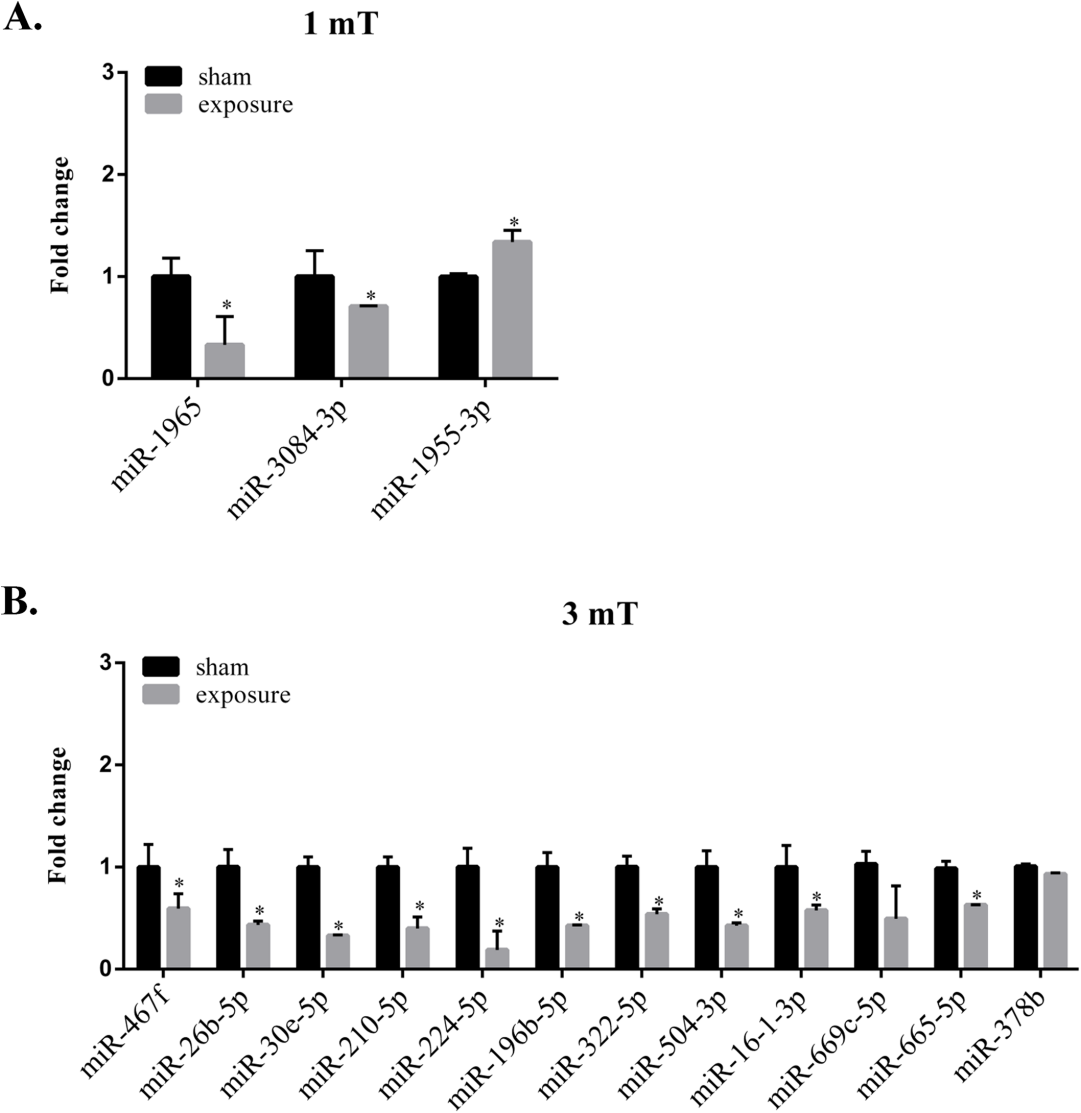
Real-time PCR verification of microarray data. A. The differentially downregulated genes detected in GC–2 cells at a magnetic field intensity of 1 mT were validated using real-time PCR. miR–1965 was the most altered miRNA at a magnetic field intensity of 1 mT. B. The differentially downregulated genes detected in GC–2 cells at a magnetic field intensity of 3 mT were validated using real-time PCR. A magnetic field intensity of 3 mT most affected the expression of miR-224-5p. The expression levels of miR-26b-5p, miR-30e-5p, miR-210-5p, miR-224-5p, miR-196b-5p, miR-504-3p and miR-669c-5p significantly differed between the ELF-EMF and the sham groups (Fold change > 2.0).

To validate the miRNA array data, we selected several differentially expressed miRNAs for real-time PCR quantification. According to the real-time PCR results, the expression of the selected miRNAs was consistent with the Affymetrix array data for the GC–2 cells (Fig 4A and 4B), suggesting that the microarray data were reliable for further analysis. Specifically, the miRNAs showing the greatest changes in expression were miR–1965 and miR-224-5p at magnetic field intensities of 1 mT and 3 mT, respectively, and these miRNAs were also downregulated in the microarray data. Among the selected miRNAs, the expression levels of miR-26b-5p, miR-30e-5p, miR-210-5p, miR-224-5p, miR-196b-5p, miR-504-3p and miR-669c-5p significantly changed compared with the sham group (Fold change > 2.0). These results suggested that these miRNAs might represent core miRNAs that play key roles in the effects of ELF-EMFs.

### Network analysis

**Fig 5:**
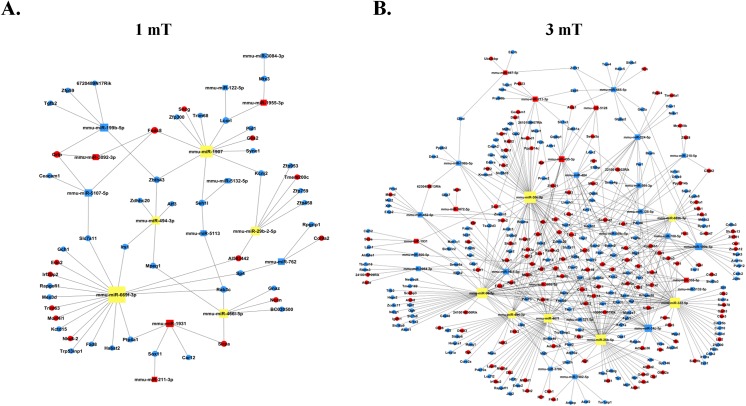
Schematic representation of a combination of the most significant networks following 50 Hz ELF-EMF exposure. A. miRNA-gene-net in the GC–2 cell line between the sham and exposure groups at a magnetic field intensity of 1 mT. B. miRNA-gene-net in the GC–2 cell line between the sham and exposure at a magnetic field intensity of 3 mT. A red dot indicates upregulated genes, a blue dot indicates downregulated genes, and a lilac dot indicates connection genes.

To identify the cellular functions associated with ELF-EMF exposure-mediated changes at the level of miRNA expression, a network analysis was performed to predict putative miRNAs and their target genes. The network analysis was carried out using the Target Scan software. A red dot indicates upregulated genes, and a blue dot indicates downregulated genes. We found that miR-211-3p, miR-494-3p, miR-669f-3p and miR–1907 may represent important miRNAs in GC–2 cells at a magnetic field intensity of 1 mT (Fig 5A). However, miR-30e-5p, miR-210-5p, miR-224-5p, miR-196b-5p, miR-504-3p, miR-669c-5p and miR-455-3p may be closely related to the epigenetic mechanism associated with ELF-EMF exposure at a magnetic field intensity of 3 mT (Fig 5B).

### Validation of the expression of selected miRNAs following 50 Hz ELF-EMF exposure

**Fig 6:**
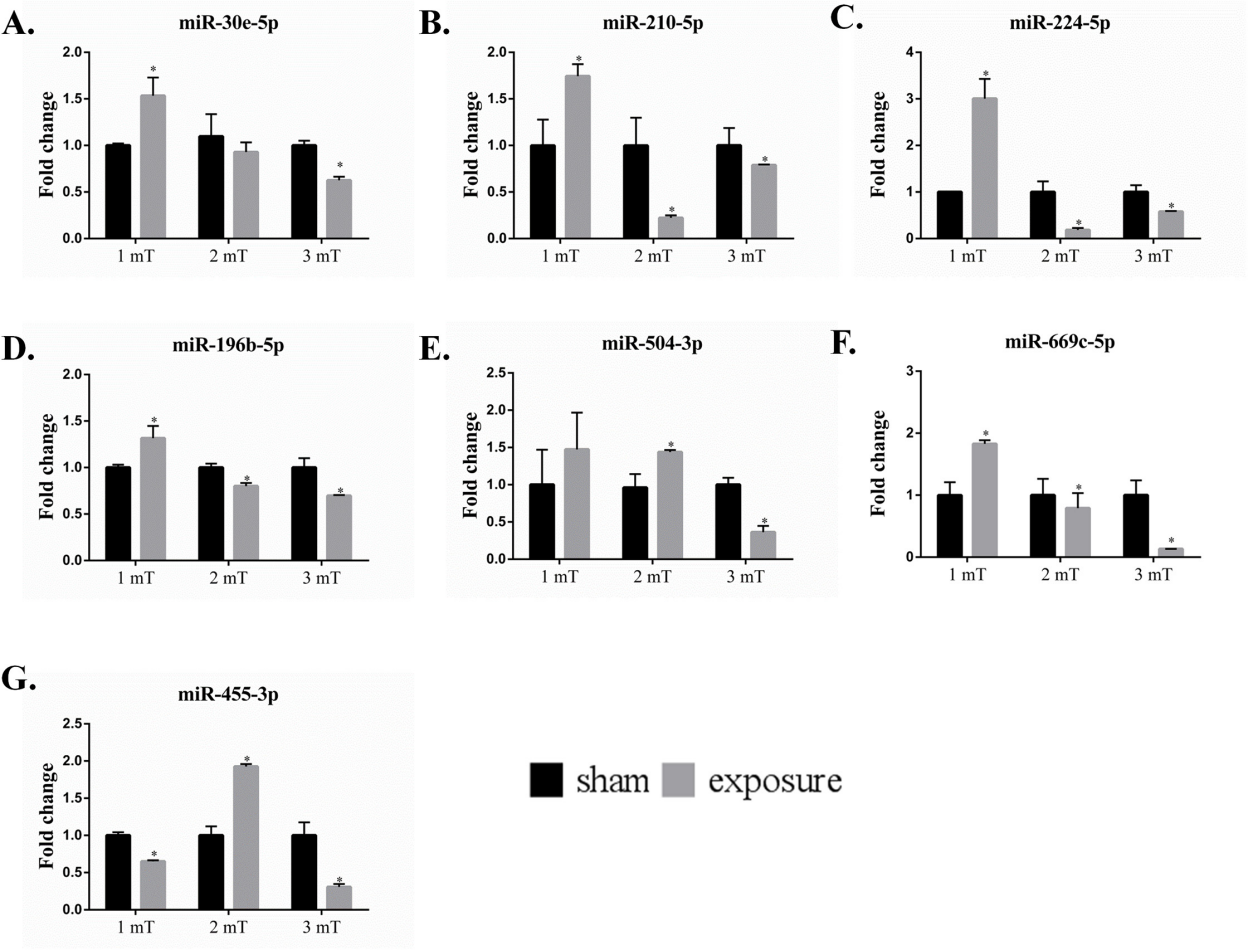
Validation of the expression of selected miRNAs following EMF exposure. The expression levels of miR-30e-5p, miR-210-5p, miR-224-5p miR-196b-5p and miR-669c-5p were significantly higher in response to a magnetic field intensity of 1 mT but were significantly lower in response to magnetic field intensities of 2 mT and 3 mT than in the sham-exposure group (A, B, C, D and F). miR-504-3p and miR-455-3p were downregulated at magnetic field intensities of 1 mT and 3 mT and upregulated in response to a 2 mT electromagnetic field (E and G).

Following the network analysis and confirmation of the miRNA array data via real-time PCR, we examined the effect of the magnetic field intensity on the expression levels of miR-30e-5p, miR-210-5p, miR-224-5p, miR-196b-5p, miR-504-3p, miR-669c-5p and miR-455-3p (Fig 6). The ELF-EMF affected the expression of all miRNAs. Notably, the expression levels of miR-30e-5p, miR-210-5p, miR-224-5p miR-196b-5p and miR-669c-5p were significantly upregulated at a magnetic field intensity of 1 mT, but significantly downregulated at 2 mT and 3 mT compared with the sham group. miR-504-3p and miR-455-3p were downregulated at magnetic field intensities of 1 mT and 3 mT, but upregulated in response to a 2 mT electromagnetic field. We have used a two-way ANOVA and simple t-test to analysis our data, our results confirmed that EMF did not show dose-dependent effects at different magnetic intensities (1, 2 and 3 mT).

### Analysis of 50 Hz ELF-EMF-specific putative miRNA target genes and functions

To obtain insights into the possible roles of the most evidently altered miRNAs, we systematically analyzed the current literature on the most strongly affected miRNA target genes and their biological functions. miRNAs reportedly function by inhibiting the expression of target genes. Therefore, we screened the target genes of the miRNAs using Target Scan (http://www.targetscan.org/), as summarized in Tables 3 and 4. This analysis indicated that miR-210-5p is an important miRNA involved in cell proliferation and the induction of apoptosis, while miR-224-5p is an oncogene that might induce platinum resistance in ovarian papillary serous carcinoma (OPSC) (Table 3). These miRNAs are thought to play an important role in GC–2 cells following ELF-EMF exposure.

**Table 3:** Proposed functions of the most evidently changed miRNAs following exposure to EMF radiation.

microRNA	Proposed function	References
miR-30e-5p	Epithelial mesenchymal transition	[44]
miR-210-5p	Apoptosis	[45]
miR-224-5p	Oncogene and induction of platinum resistance	[46]
miR-196b-5p	Impairment of HR repair	[47]
miR-504-3p	Reduction of p53 expression	[48]
miR-669c-5p	Regulation of glutathione metabolism	[49]

**Table 4:** Differentially expressed miRNAs following EMF irradiation and their predicted target genes.

miRNA	Predicted target genes
miR-26b-5p	Mxi1, Bod1, Gpr146, Srgap1, Plod2, Ralgds, Vldlr, Hectd2, Rgs9, Ube2h h, Slc4a4, Trp53inp1, Cdh2, Enc1, Kcnj2, Man2a1, Plxna2, Rb1, Adam19, Ccnd2, Cdh11
miR-30e-5p	Rgs9, Dact1, Slc6a9, Gtf2h1, Hmgb3, Serpine1, Plxna2, Rgs2, Sox11, Adam19, Gda, Gdnf, Ednra, Elavl3, Ier2, Irs1, Klf9
miR-210-5p	Hectd2, Cited2, Mrps18b, Snx30, Zfp28
miR-224-5p	Gtpbp2, Slc4a4, Enc1, Fosb, Serpine1, Ptx3, Dnm1, Nr4a1, Timm8a1, Sort1, Rnd3, Zdhhc20, Mbnl3, Rps24
miR-196b-5p	Dnm3, Rbpms, Tsc22d3, Sox11, Chrd, Ppp6r2, 6230409E13Rik, Tanc2
miR-504-3p	Cxcl12, Fmn1, Per1, Fosb, Elavl3, Sema4g, Wisp1, Bcam, Tanc2
miR-669c-5p	Mxi1, Bod1, Gm949, Gpr146, Srgap1, Plod2, Ralgds, Vldlr, Hectd2, Rgs9, Ube2h, Slc4a4, Trp53inp1, Cdh2, Enc1, Kcnj2, Man2a1, Plxna2, Ptx3, Rb1, Adam19

### GO term and KEGG pathway annotation based on the miRNA expression profile

**Fig 7:**
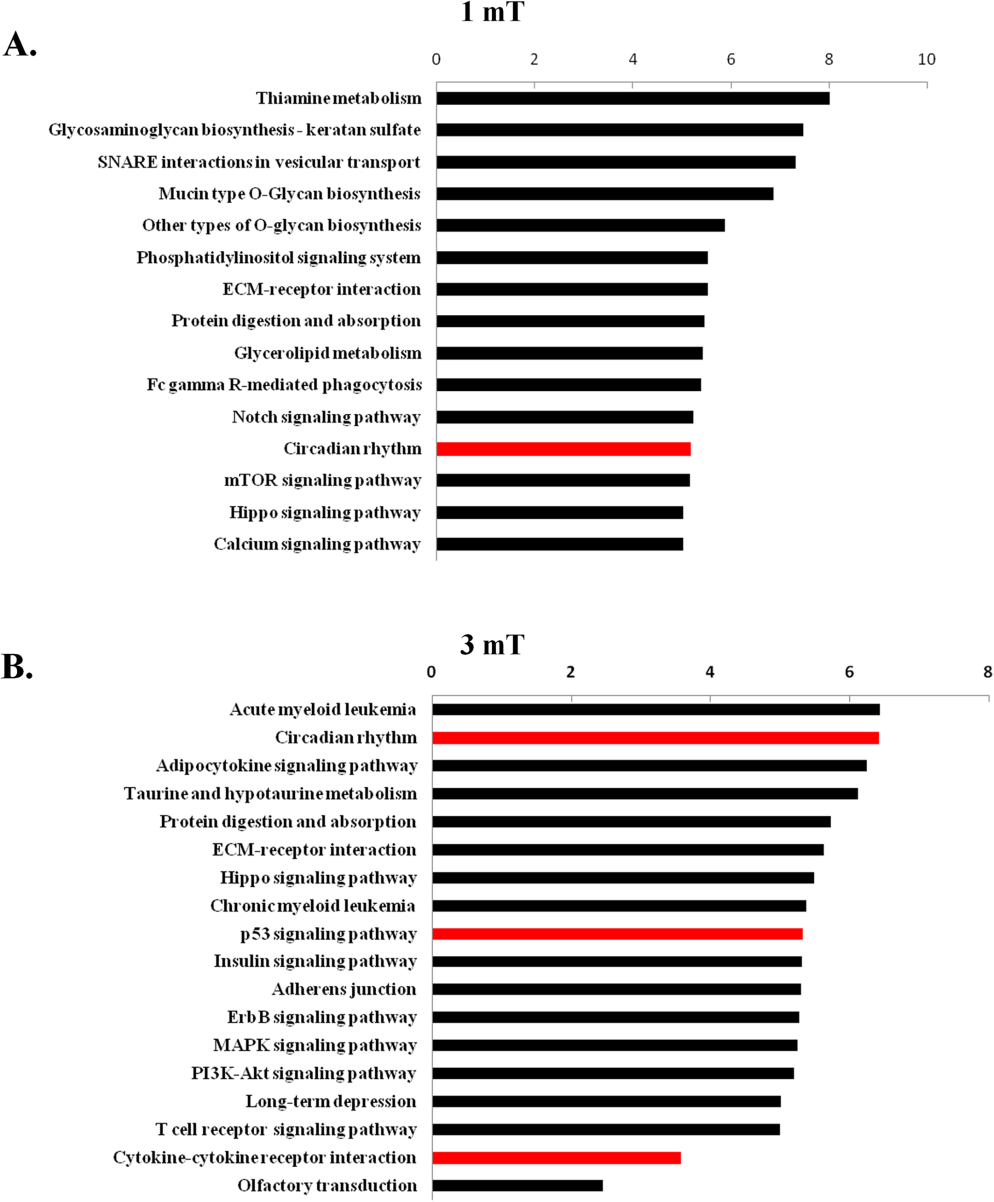
Pathway analysis based on miRNA target genes. A. Predicted significant signaling pathways targeted by altered miRNAs at a magnetic field intensity of 1 mT. B. Predicted significant signaling pathways targeted by altered miRNAs at a magnetic field intensity of 3 mT. The vertical axis corresponds to the pathway category and the horizontal axis to the enrichment of pathways.

The functional analysis of the miRNA target genes according to KEGG pathways revealed that 15 predicted significant signaling pathways showed alterations at a magnetic intensity of 1 mT, and 19 exhibited changes at 3 mT (Fig 7A and 7B). Many of these signaling pathways have been shown to participate in the miRNA-mediated regulation of signaling pathways, including the mTOR signaling pathway, circadian rhythms, the p53 signaling pathway, long-term depression and the MAPK signaling pathway. We found that core miRNAs might regulate circadian rhythms, cytokine-cytokine receptor interactions and the p53 signaling pathway, and these pathways could play significant roles in the biological effects of ELF-EMFs.

## Discussion

The present study clearly indicated that intermittent exposure to a 50 Hz ELF-EMF did not alter the cell viability, cell cycle or apoptosis of GC–2 cells. However, a 50 Hz ELF-EMF did affect the expression of miRNAs in the GC–2 cell line. A further analysis of the affected miRNAs and putative target genes indicated that circadian rhythms, cytokine-cytokine receptor interactions and the p53 signaling pathway represented important mechanisms of action that were epigenetically regulated in GC–2 cells following 50 Hz ELF-EMF exposure. Thus, we showed, for the first time, that miRNA regulation might play a key role in the biological effects of a 50 Hz ELF-EMF.

In present study, the used intermittent exposure with 5 min on and 10 min off in the present study was first studied by Ivancsits S [21] and then replicated in later studies [5, 22, 23], which showed that it had more powerful effects than continuous exposure to induce DNA strand breaks. Subjecting cells continuously to a constant field may induce adaptive mechanisms, protecting the genome from harmful influences. It hs reported that DNA damage may be involved in cell proliferation, apoptosis as well as cell cycle [24, 25], and few studies have been conducted to apply this intermittent exposure to GC–2 cells. Meanwhile intermittent exposure can imitate the regular change of environmental conditions which might interfere with such mechanisms and lead to DNA impairment. Therefore, it is meaningful to unveil the potential adverse effects of 50 Hz ELF-EMF on GC–2 cells using this exposure mode.

A large body of evidence has confirmed that ELF-EMFs can affect the male reproductive system by inducing spermatogenic cell apoptosis, decreasing the numbers of spermatogenic cells and significantly increasing abnormalities in cells [26]. However, the underlying molecular mechanism of this influence remains unclear. miRNAs have been confirmed to be able to regulate gene expression related to proliferation, the cell cycle and apoptosis. The development and progression of cancer have been linked to changes in miRNA expression [27]. Yuan et al. reported that miR-34b/c and miR-449a/b/c are necessary for spermatogenesis [28]. miR-142-3p plays a key role in the pathogenesis of TGCTs by repressing PTPN23 expression [29]. miRNAs are also involved in the development of the immune system and the pathogenesis of chronic inflammatory disorders [30, 31]. In addition, the aberrant expression of specific miRNAs is linked to certain male reproductive disorders [32, 33]. Therefore, we speculated that miRNA regulation might be associated with ELF-EMFs.

The CCK–8 assay was used to evaluate cell viability. This assay can also indirectly reflect cell numbers and cell proliferation. Our results showed that exposure to 50 Hz ELF-EMF did not influence the proliferation of GC–2 cells. Similar to our findings, Ma et al. found that intermittent exposure to ELF-EMFs (0.5 mT, 1 mT and 2 mT for 3 days, 5 min on / 10 min off) did not affect the cell cycle or cell proliferation of embryonic neural stem cells [5]. However, Huang et al. found that exposure to ELF-EMFs (1.5 mT, 60 Hz ELF-EMF) for 144 h inhibited HaCaT cell growth [34]. Therefore, the different exposure modes (continuous or intermittent), magnetic intensities and exposure times may lead to different results.

Many studies have shown that apoptosis and the cell cycle may play a key role in regulating the process of proliferation [35]. We did not observe significant differences in apoptosis and the cell cycle between the sham and exposure groups. Nikolova found that intermittent exposure to ELF-EMF (50 Hz, 2 mT, 5 min on / 30 min off) did not affect the cell cycle or cell proliferation in ES-derived neural progenitor cells, which was similar to our findings. Additionally, the morphology of GC–2 cells did not significantly differ between the sham and exposure groups in our study. These findings indicated that a 50 Hz ELF-EMF did not affect the proliferation, apoptosis or cell cycle of GC–2 cells under our experimental conditions.

Although the 50 Hz ELF-EMF did not significantly affect cell growth, apoptosis or the cell cycle, it clearly affected miRNA expression. To the best of our knowledge, previous studies have not addressed the biological effect of ELF-EMF-induced changes in miRNA expression in the GC–2 cell line. We also applied real-time PCR to confirm the differential expression of miRNAs following 50 Hz ELF-EMF exposure. The expression of selected miRNAs was consistent with the Affymetrix array data. We observed that miR-494-3p was the most highly upregulated miRNA among the differentially expressed miRNAs. miR-494-3p may act as an oncogene by targeting genes related to the cell cycle and apoptosis [36]. In addition, miR-494-3p can suppress cell proliferation and induce senescence, and the overexpression of miR–494 can lead to the downregulation of Cdk6 and enhance G1 arrest [37, 38]. We further applied a network analysis to predict putative miRNA target genes and their biological functions. Our analyses showed that many of the predicted miRNA target genes were involved in critical cellular pathways. A recent study found that each miRNA can regulate hundreds of target genes, and each mRNA can also be regulated by many miRNAs [39]. Ccnd2, a cell-cycle associated gene, is a potential target of miR-322-5p, miR-328-5p, miR-378b, miR-669b-5p and miR–206, whose expression are altered by exposure to ELF-EMFs [40, 41]. In our study, the expression of miR-322-5p and miR-378b was down-regulated at a field intensity of 3 mT, and this might upregulate the expression of Ccnd2, Whose aberrant expression could lead to abnormal cell proliferation. In addition, Slc7a11 which might play an important role in conveying resistance to apoptosis is a potential target of miR-30e-5p [42]. The downregulation of miR-30e-5p might alter the expression of Slc7a11 and might induce apoptosis of GC–2 cells. Furthermore, Usp45 is also potential target of miR-30e-5p which is a regulator of XPF-ERCC1 crucial for efficient DNA repair [43]. Therefore, we speculate that miRNAs might induce some significant effects via target genes and long-term ELF-EMF exposure might induce alterations in cell growth or regulate some key signaling pathway. Such claims need further investigation.

To obtain insights into the potential functions of the differentially expressed miRNAs, we systematically analyzed the current literature on the associated target genes and biological functions. This analysis indicated that miR-30e-5p is related to the epithelial mesenchymal transition [44]. miR-210-5p is involved in cell proliferation and the induction of apoptosis [45]. miR-224-5p is an oncogene and can induce platinum resistance in OPSC [46]. miR-196b-5p can target c-myc and Bcl–2 expression to inhibit proliferation and induce apoptosis in endometriotic stromal cells [47]. TFF1 activates p53 by downregulating miR–504 in gastric cancer [48], and miR-669c-5p is associated with the regulation of glutathione metabolism [49]. These miRNAs might play an important role in GC–2 cells following exposure to a 50 Hz ELF-EMF.

To obtain insights into the potential functions of the altered miRNAs, GO term and KEGG pathways were applied to their target genes. The enrichment ranking of signaling pathways showed that circadian rhythms, cytokine-cytokine receptor interactions and the p53 signaling pathway may be involved in the biological effects of ELF-EMFs. This functional identity, which was revealed based on different bioinformatic interpretations, confirmed that the effects of miRNAs on GC–2 cells exposed to 50 Hz ELF-EMF involve the activation of signaling pathways.

In summary, we demonstrated that intermittent exposure to a 50 Hz ELF-EMF can induce changes in miRNA expression. However, based on the lack of detectable changes in cell proliferation, the cell cycle and apoptosis, we speculate that long-term exposure to ELF-EMFs may negatively impact human health. Our findings suggest that miRNAs can be used as biomarkers of early-stage ELF-EMFs exposure, and the miRNA-mediated regulation of signaling pathways might play key roles in the biological effects of ELF-EMFs. Such claims require further investigation.
